# Demographic Data, Risk Factors, and Disease Burden of HS Patients in Lithuania at a Reference Center

**DOI:** 10.3390/healthcare12181849

**Published:** 2024-09-14

**Authors:** Tadas Raudonis, Austėja Šakaitytė, Tomas Petras Vileikis, Vitalij Černel, Rūta Gancevičiene, Christos C. Zouboulis

**Affiliations:** 1 Clinic of Infectious Disease and Dermatovenereology, Institute of Clinical Medicine, Faculty of Medicine, Vilnius University, M. K. Čiurlionio g. 21, 03101 Vilnius, Lithuania; austeja.sakaityte@gmail.com (A.Š.); tomas.vileikis97@gmail.com (T.P.V.); ruta.ganceviciene@gmail.com (R.G.); 2 European Hidradenitis Suppurativa Foundation e.V., 06847 Dessau, Germany; christos.zouboulis@t-online.de; 3 Faculty of Medicine, Vilnius University, M. K. Čiurlionio g. 21, 03101 Vilnius, Lithuania; vitalijus@bardai.lt; 4 Departments of Dermatology, Venereology, Allergology and Immunology, Dessau Medical Center, Brandenburg Medical School Theodor Fontane and Faculty of Health Sciences Brandenburg, 06847 Dessau, Germany

**Keywords:** hidradenitis suppurativa, demography, risk factors, cost of illness

## Abstract

Background: Hidradenitis suppurativa (HS) diagnosis often faces a global delay of 7.2 years due to factors like lack of recognition, stigma, and socioeconomic barriers. Limited effective therapies and frequent exacerbations impact patients’ quality of life, posing a significant burden on healthcare systems. Methods: HS patients were assessed according to European Hidradenitis Suppurativa Foundation (EHSF) Registry questionnaire guidelines at various stages of the disease and treatment. Results: The study included 49 patients; 57.14% (n = 28) of them were male. The average age of the subjects was 39.91 ± 13.665 years; the average BMI was 27.84 ± 7.362. A total of 59.18% (n = 29) were active or previous smokers. There were statistically more male smokers than female (*p* < 0.01). Average disease onset was 25.71 ± 13.743 years; the mean time to diagnosis was 5.2 ± 7.607 years. A total of 70.2% (n = 33) were previously misdiagnosed. Subjects had 6.17 ± 6.98 painful days over the preceding 4 weeks. The average intensity of pain according to the visual analogue scale (VAS) was 5.60 ± 3.36 points. The mean dermatology life quality index (DLQI) at baseline was 8.9 ± 7.436. Conclusions: The research revealed delayed diagnoses, especially for females. Smoking was linked to higher Hurley stages, with a prevalence among male smokers, and HS had a substantial impact on patients’ quality of life.

## 1. Introduction

Hidradenitis suppurativa (HS) is a persistent, inflammatory, recurring, and incapacitating skin disorder affecting hair follicles. It manifests with painful, deep-seated, inflamed lesions in the regions of the body that bear apocrine glands [1]. HS typically begins after puberty, with the most common age of onset being between 20 and 29 years [2]. However, HS can affect individuals of all ages, including children and older adults [3].

The development of HS is influenced by a combination of factors, including genetic predispositions, environmental factors (such as smoking and obesity), hormonal factors, and immune dysfunction [4]. There is a growing understanding that the interaction between endogenous and exogenous factors contributes to activation of the innate immune system, primarily leading to inflammation around hair follicles [5]. This inflammatory response causes hyperkeratosis and hyperplasia of the follicular epithelium, particularly in the infundibulum. Consequently, follicular occlusion occurs, resulting in blockage of the follicle [6]. Subsequent dilation and rupture of the hair follicle triggers a strong inflammatory immune response, characterized by the recruitment of various immune cells including neutrophils, macrophages, B-cells, and Th1 and Th17 cells into the affected skin areas. This immune cell activity leads to the formation of inflammatory nodules or abscesses [4]. During this immune response, proinflammatory cytokines such as tumor necrosis factor-alpha (TNF-α), interleukin (IL)-1β, IL-6, CXC chemokine ligand (CXCL)/IL-8, IL-12p70, IL-23p40, IL-17A, and IL-36 are produced [4,7,8,9,10]. These cytokines play a significant role in the immune dysregulation seen in both the acute and chronic stages of HS [11]. The presence of these proinflammatory cytokines, particularly those associated with the Th17 immune response, is considered to be a prominent feature of HS [4,7,8,9,10]. Recent research has provided additional evidence supporting an autoinflammatory mechanism in HS. One notable finding is the increased formation of neutrophil extracellular traps (NETs) observed in HS skin. The immune reactions triggered by neutrophils and NET-related antigens have been linked to heightened immune dysregulation and inflammation in the disease [12]. As the inflammatory process progresses, tissue scarring becomes more prominent [13,14,15,16]. The development of scarring and sinus tracts has been associated with the presence of metalloproteinase-2, tumor growth factor (TGF)-β, and intercellular adhesion molecule (ICAM)-1. Additionally, it is speculated that specific components of the microbiome may enhance TGF-β and ICAM-1 signaling, contributing to these processes [13,14].

One of the most important environmental factors in HS pathophysiology is smoking as around 90% of individuals diagnosed with HS are either current or former smokers, and smoking is believed to contribute to the development and progression of the disease through various mechanisms. One such mechanism involves the pro-inflammatory role of polycyclic hydrocarbons, which are produced as a result of cigarette combustion [17]. Nicotine may also impact the propagation of bacteria and the formation of biofilms in various ways. For instance, it can promote the initial attachment and accumulation of these microorganisms, modify the anti-inflammatory lipid film on the skin, and facilitate the colonization of pathogenic bacteria in the affected area [18,19,20,21,22]. HS is considered to be more of a dysbiosis rather than an infection as early lesions exhibit a reduction in regulatory T-cells, and the absence of biofilms and bacteria is noticeable [23]. In fact, bacterial colonization and the formation of biofilms are observed as late-stage events in HS, whereas early lesions exhibit an absence of bacteria and lack a normal biofilm structure [24,25,26,27,28].

There is a positive association between increased body mass index (BMI) and the presence and severity of HS [29,30]. Approximately 50% of individuals with HS are classified as obese, and around 40% of HS patients show signs of metabolic syndrome. Obesity is thought to play a role in the pathogenesis of HS through mechanisms such as subclinical inflammation, metabolic alterations, and heightened friction in skin folds. Additionally, there is suspicion of a contribution from sex hormones, particularly the androgen receptor, in development of the disease [31].

Individuals affected by HS encounter a substantial time lapse, which is estimated at 7.2 years globally, between the onset of the disease and its diagnosis. This delay is influenced by various factors, such as doctors’ lack of recognition, embarrassment and stigma attached to the condition, and socioeconomic obstacles [32,33,34]. Nevertheless, achieving consistent symptom control and complete resolution of lesions is challenging and frequently insufficient, potentially resulting in escalated healthcare expenses [35,36]. The limited availability of effective therapies and frequent exacerbations present a difficult obstacle in treatment, resulting in a detrimental effect on quality of life [37,38]. In addition, given the painful nature of the lesions, their occurrence in sensitive areas, and the presence of drainage, unpleasant odor, and resulting scarring, HS can significantly impact a person’s psychological and social well-being. As HS significantly compromises the quality of a patient’s life, it is crucial that diagnosis should be identified without delays to decrease the burden of the disease on patients and the healthcare system [39].

The aim of this study was to evaluate the main demographic data of HS patients presenting to a reference center, Centre of Dermatovenereology in Lithuania, along with risk factors and disease burden.

## 2. Materials and Methods

HS patients presenting to a reference center, Vilnius University Hospital Santaros Klinikos Centre of Dermatovenereology in Lithuania, from March 2021 to June 2023 were assessed according to European Hidradenitis Suppurativa Foundation (EHSF) Registry questionnaire guidelines at various stages of the disease and treatment. The demographic data of patients, such as age, gender, body mass index (BMI), education level, skin phototype, hair structure (i.e., straight, wavy, and curly), and occupation, were recorded. We also documented smoking habits and their association with the severity of HS according to Hurley stages, age of patients at diagnosis, disease duration, diagnostic delay, most common misdiagnoses, pain intensity according to the visual analogue scale (VAS), and anxiety score using a 0–10 scale. Data were processed using MS Excel and IBM SPSS 26.0. Normal distribution of the data was assessed using a Shapiro–Wilk test. Homogeneity of the groups was evaluated using a chi-square test. Differences were considered statistically significant when the value was below the significance interval of 0.05 (*p* < 0.05).

The research received approval from the Vilnius Regional Biomedical Research Ethics Committee (ethical approval number 2021/2-1310-793; approval date 23 February 2021). All participants provided written informed consent prior to their involvement in the study.

## 3. Results

### 3.1. Demographics

**Figure 1:**
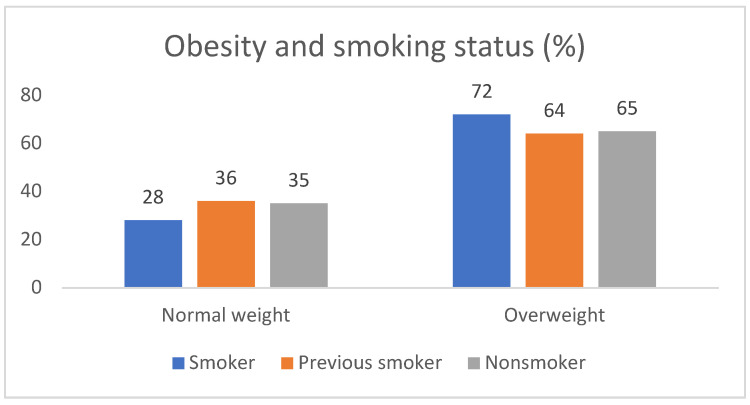
Smoking and obesity association among HS patients.

The study included 49 patients of which 57.14% (N = 28) were male. All patients were Caucasian. The average age of the subjects was 39.91 ± 13.665 years; the age range was 19–74 years; the average BMI was 28.44 ± 6.142; the average waist circumference was 91.85 cm; 30.61% (N = 15) were overweight and 36.73% (N = 18) were obese. There was no statistical correlation between gender and BMI variables. There was a statistically significant correlation between obesity and smoking status variables (r = 0.673, *p* < 0.01) (Figure 1).

A total of 51.02% (N = 25) of subjects had higher education. Disease severity does not depend on hair structure (*p* = 0.467) or skin phototype (*p* = 0.631). The data are distributed according to a normality curve; the groups are homogeneous (Table 1).

Average disease onset was at 25.71 ± 13,743 years; patients sought medical care at 28 years, and the mean time to diagnosis was 5.2 ± 7.607 years. Female patients were diagnosed significantly later than males; on average 6.5 years versus 4.2 years (*p* = 0.01) (Table 1).

A total of 70.2% (N = 33) were previously misdiagnosed; 51.51% (N = 17) of patients were diagnosed with a furuncle (Table 1). A total of 87.75% (N = 43) of patients were diagnosed with HS by a dermatovenereologist.

### 3.2. Smoking

A total of 36.73% (N = 18) of subjects were active smokers and 22.44% (N = 11) were previous smokers with an average of 26.56 pack-years. A total of 40.81% (N = 20) of participants were nonsmokers. Among 12 Hurley III patients, 91.66% (N = 11) were smokers; a statistically significant correlation was found (r = 0.659, *p* < 0.01) between these two variables. The distribution between smokers and nonsmokers with different Hurley stages was statistically significant as well (*p* < 0.01). There are statistically more male smokers than female (*p* < 0.01), with 33.33% (n = 7) of women and 78.57% (n = 22) of men being smokers.

### 3.3. Pain

On average, subjects had 6.17 ± 6.98 painful days over the preceding 4 weeks. The average pain intensity score according to the VAS scale was 5.60 ± 3.36. Females indicated that their average pain intensity score was 5.42 points, while males indicated an average pain intensity score of 5.74 (Table 2). A total of 30.61% (N = 15) had persistent pain and 69.38% (N = 34) had intermittent pain. Male patients statistically significantly complained more often with intermittent pain (*p* = 0.003). A total of 24.48% (N = 12) of subjects claimed that HS exacerbation was caused by pressure or mechanical friction.

### 3.4. Disease Burden and Quality of Life

A total of 36.73% (N = 18) were hospitalized for HS with an average length of 19.5 days of hospitalization; of them, 66.66% (N = 12) were males and 61.11% (N = 11) had severe HS with Hurley stage III. A total of 26.53% (N = 13) of all subjects admitted that HS affected their professional career. A total of 24.48% (N = 12) missed work due to HS; on average, over 6 months, patients missed 20.5 working days. A total of 76.92% (N = 10) of them had a higher education diploma.

Patients were worried about their illness; the average score of their anxiety level was 6.5 ± 2.586 points out of 10. Male patients worried about the disease more often; they rated their anxiety level at 6.84 vs. 6.04 for females (*p* = 0.014). Age has a slight correlation with anxiety level—the younger the patient, the higher the anxiety score (r = 0.231, *p* = 0.002). Patients rated the disease impact on quality of sleep with an average of 2.68 ± 3.060 points out of 10. A total of 77.55% (N = 38) marked their anxiety level > 5; all these patients had worse sleep quality than subjects with an anxiety score of <5 (*p* = 0.02).

**Figure 2:**
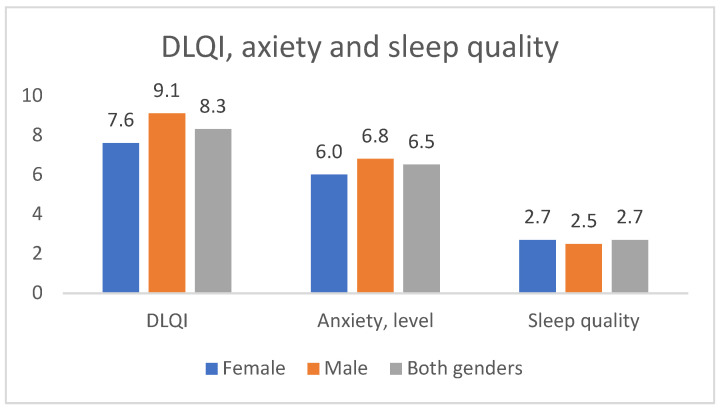
Dermatological quality of life, anxiety levels, and sleep quality of HS patients.

The mean dermatology life quality index (DLQI) at baseline was 8.30 ± 7.461 for all subjects (Figure 2). A total of 32.65% (N = 16) patients had a DLQI of >10; 62.5% (N = 10) of them were males. A total of 93.75% (N = 15) had a BMI > 25 (*p* < 0.01), and their anxiety level score was 7.5 points.

## 4. Discussion

### 4.1. Demographics

Studies show that in North American and European populations, HS is more prevalent among females, with a female-to-male ratio of 3:1 [30,40,41]. However, a different pattern emerges in South Korea, where the female-to-male ratio is approximately 1:2 [42,43]. The highest prevalence of HS is observed in individuals in their fourth and fifth decades of life; however, this chronic skin condition commonly emerges during young adulthood, with a notable spike in new diagnoses occurring among those aged 18 to 29 years in the USA [30,40,41,44]. In our study, which included 49 patients, 57.14% were male, and the average age of the subjects was 39.91 ± 13.665 years. The hypothesis claims that HS onset often coincides with or shortly follows puberty [32]. An association has been noted between HS and a lower socioeconomic status, which could be partially linked to an increased prevalence of HS risk factors within this demographic group, or alternatively, it could arise as a result of HS itself [45]. A retrospective Dutch study of 846 patients found that despite the fact that HS is more common in women, men tend to suffer from a more severe form of the disease [46].

The prevalence of HS continues to be a topic of considerable debate as estimates for Europe and North America vary between 0.05% and 4.1%. These variations are influenced by the demographics of the populations under study and the methodologies employed [47,48]. One of the meta-analyses of HS global prevalence determined that the overall HS prevalence rate reaches 0.3%. Subgroup analysis revealed that Europe had the highest prevalence at 0.8%, in comparison to rates of 0.2% in the USA, Asia–Pacific, and South America [49]. In the United States, the prevalence of HS varies among different ethnic groups. African Americans tend to have the highest prevalence, followed by Caucasians, while Hispanics and Asians have lower prevalence rates [50]. Within Asian populations, individuals with darker skin tones are disproportionately affected compared to those with lighter skin [51,52].

### 4.2. Smoking and Obesity

HS is highly influenced by two environmental factors: smoking and obesity. A thorough analysis encompassing 6174 HS cases and 24,993 controls revealed a significant correlation. The odds ratio (OR) for current smoking and HS stood at 4.34, while for obesity and HS, the OR was 3.45 [53]. Increased smoking pack-years and elevated BMI have both been associated with more pronounced disease severity [46]. While obesity and smoking are acquired disease factors, their role in disease development might be connected to the suppression of Notch signaling. Downregulation of the Notch pathway has been documented in individuals who smoke, and this pathway also plays a role in regulating metabolism. The disruption of this pathway results in an impaired innate immune response, which could provide insight into the immune dysregulation believed to be a key factor in both the development and clinical manifestation of HS [54].

According to Konig et al., the prevalence of active cigarette smokers among HS patients was close to 90% [55]. Based on these results, Breitkopf and his team discovered that the occurrence of smoking behaviors among male and female patients was 85% and 84%, respectively [56]. Dessinotti et al. revealed that a mere 16% of HS patients had not smoked at any point [57]. Our analysis yielded comparable results, with 59.18% of our participants having a history of active or past smoking. Moreover, a statistically significant association was discovered between being diagnosed with Hurley III and having a smoking history as 91.66% of Hurley III patients were smokers.

However, the status of being an ex-smoker does not appear to exhibit a distinct correlation with this condition [58]. Taking these factors into account, active tobacco smoking could be regarded as a potential risk factor for HS [59]. Certainly, this condition is linked to a twofold rise in the risk of developing HS [60]. Conversely, cigarette smoking could potentially be a result of the disease as patients might turn to smoking as a means to alleviate the anxiety and depression often connected with HS [61,62]. As previously indicated, cigarette smoking might also play a role in providing insight into the gender distribution of HS [63]. The connection between tobacco smoking and disease severity among HS patients has also been evaluated. Sartorius at al. observed that individuals who smoked exhibited greater disease severity, as measured by the modified Hidradenitis Suppurativa Score (mHSS), when compared to nonsmokers [64]. The impact of cigarette smoking on disease management is noteworthy as research revealed that not smoking was associated with higher chances of responding positively to initial treatment and a greater frequency of self-reported remission from HS [65,66].

Between 50% and 75% of HS patients fall into the overweight or obese category [67]. In our study, the average BMI was 27.84 ± 7.362. Among participants, 30.61% were categorized as overweight and 36.73% fell into the obese category. Multiple studies have shown a correlation between higher BMI and increased severity of HS. In two case–control studies, it was observed that for every unit increase in BMI, there was a 1.12-fold higher risk of developing HS [68]. Other research examining the impact of weight loss on HS severity revealed notable findings: a substantial decrease in the proportion of patients experiencing HS symptoms (up to 35%) after weight loss and a decrease in the number of affected anatomical sites from 1.93 to 1.22 [69,70]. Therefore, it can be reasonably deduced that bariatric surgery, which is an intervention for severe obesity, holds the potential to alleviate the impacts of inflammatory skin conditions like HS [71]. In recent developments, semaglutide and liraglutide, which are both recognized as glucagon-like peptide-1 receptor analogs (GLP-1RA) and authorized for managing diabetes and obesity, have demonstrated efficacy in not only causing notable weight loss but also in reducing several obesity-related comorbidities [72,73]. Two case reports demonstrated a decrease in the clinical severity of HS following the administration of liraglutide, accompanied by significant weight loss [74,75]. In a phase 3 trial involving individuals with obesity or who were overweight, the utilization of tirzepatide, which is a dual agonist targeting both GLP-1 receptors and another incretin, gastric inhibitor polypeptide (GIP), led to weight reduction of as much as 20.9% over an 18-month period [76]. Furthermore, the antidepressant bupropion showed a dose-dependent decrease in weight among individuals categorized as obese or overweight [77,78]. The link between BMI and HS severity could be elucidated by the higher count of skin folds in individuals with excess weight, fostering follicular blockage and elevating mechanical friction that can initiate the rupture of enlarged follicles [69]. Obesity induces a mild inflammatory state within the body, substantiated by elevated levels of circulating proinflammatory cytokines observed in these individuals [70]. The heightened quantity of adipose cells, which function as endocrine tissues capable of releasing inflammatory cytokines, is viewed as a contributing factor to the persistent inflammatory state observed in HS [79].

### 4.3. Duration until a Diagnosis Is Determined

The difficulty in diagnosing HS clinically arises from the extensive diversity in the disease’s presentation and inconsistent responses to suggested treatment plans [80]. The delay in diagnosing HS can lead to prolonged patient distress, hinder the gathering of epidemiological information, and result in inadequate health results. As a result of the resemblance of early hidradenitis suppurativa stages to other conditions, on a global scale, the average diagnostic delay ranges from 7 to 10 years [32,81,82,83]. This is similar to what we observed in our study, with the mean time to diagnosis being 5.2 ± 7.607 years. As there are no definitive tests available, the diagnosis of HS relies on clinical observation and the patient’s description of their condition [1]. Research conducted in Germany demonstrated a positive association between the duration of diagnostic delay and the number of medical practitioners consulted by the patient prior to receiving a HS diagnosis. Consequently, individuals with the most prolonged HS diagnostic delay had, on average, consulted nearly five physicians. Among the patients, the primary medical contacts were general practitioners, followed by dermatologists, surgeons, and gynecologists. Furthermore, individuals with delayed diagnosis encountered an average of nearly five instances of misdiagnosis. In general, there exists a positive correlation between the duration of diagnostic delay and the frequency of misdiagnoses. Common erroneous assessments included abscesses, ingrown hairs, and folliculitis. Other incorrect diagnoses comprised conditions like acne conglobata or even acne vulgaris [84]. Our study drew analogous conclusions, as 70.2% of patients were misdiagnosed, and the diagnoses included furuncle, ulcer, abscess, acne, and allergy. Ultimately, an accurate diagnosis of HS is achieved for the majority of cases by a dermatovenereologist. Misdiagnosis is correlated with factors such as non-white race, heightened disease severity, and an increased number of comorbidities [84,85]. Due to the involvement of intimate body areas, individuals with severe disease may be hesitant to reveal their symptoms or seek medical attention, influenced by feelings of shame or fear [86]. Of significant note, Kokolakis et al. revealed there was a positive correlation between the extent of diagnostic delay and the severity of HS at the point of diagnosis. As a result, patients who experienced delays in diagnosis exhibited a notably higher Hurley stage at the time of diagnosis, in contrast to those who were diagnosed earlier. Moreover, individuals who faced delayed HS diagnosis presented a greater number of comorbidities compared to those who received an earlier diagnosis [84].

This circumstance has prompted initiatives to create efficient screening methods for HS. Inquiring whether a patient has experienced episodes of abscesses in the past six months, with a minimum of two occurrences in any of the following five areas: armpits, groin, genitals, inframammary area, and unspecified locations such as the perianal area, neck, and abdomen, can result in a sensitivity of 90%, a specificity of 97%, and a positive predictive value of 96% [87]. Additionally, an alternative option is a visual questionnaire featuring illustrative images of the prevailing HS lesions [88]. Ultrasound imaging has proven valuable in enhancing understanding of the morphology and depth of lesional HS and shall be used more frequently in the future as a valuable diagnostic tool [89]. For example, it has the capacity to illustrate subclinical accumulation of fluids, elevated dermal thickness, and follicular expansion during the initial phases of HS, as well as the progression of sinus tracts as the disease advances [90,91]. The application of color Doppler ultrasound can potentially identify concealed sinus tracts, thereby contributing to more accurate treatment approaches [92]. Moreover, color Doppler can assess the vascularization of typical HS lesions, with increased vascular flow correlating with higher levels of localized pain [93].

### 4.4. Patients’ Quality of Life

Jørgensen et al. showed that there is a noticeable distinction in the average total DLQI score among the different Hurley severity groups. In their study, the mean Hurley score for people with HS was 11.9. Patients with more severe disease had a higher mean total DLQI score: 8.6 for Hurley I, 12.4 for Hurley II, and 16.1 for Hurley III. Additionally, they exhibited significantly elevated mean scores for each of the ten individual DLQI questions. Moreover, a higher overall DLQI score was linked to several factors, including younger age (below 60), unemployment, smoking, experiencing boils in the past month, having a higher boil-associated pain score, greater overall disease-related distress score, involvement of multiple anatomical regions, and specific locations such as the axillary, groin, and gluteal regions, as well as the presence of diabetes [94]. In addition, the DLQI and physician-assessed International HS Severity Score System (IHS4) scores demonstrated an upward trend with increasing disease severity according to a refined (seven-stage) Hurley classification. There was a noteworthy positive correlation between both DLQI and IHS4 scores and the progression of severity as evaluated by the refined Hurley substages [95]. In our study, patients expressed concerns about their health, with an average anxiety level score of 6.5 out of 10. Those with elevated anxiety also experienced a noticeable impact on their sleep quality. The average DLQI score at the beginning of the study was 8.9 for all subjects. Of the patients, 32.65% had a DLQI score exceeding 10; 62.5% of these were male; 93.75% had a BMI above 25, and their average anxiety level score was 7.5. Furthermore, individuals experienced an average of 6.17 ± 6.98 days of pain in the preceding 4 weeks. The average pain intensity, as assessed by the VAS scale, was 5.60 ± 3.36. Among the participants, 30.61% reported consistent or persistent pain, while 69.38% described their pain as intermittent. Male patients exhibited a statistically significant tendency to experience intermittent pain more frequently.

## 5. Conclusions

The study identified a positive correlation between smoking and the severity of HS according to Hurley stages. The research revealed delayed diagnoses, especially in females, with frequent misdiagnoses as furuncles. Patients with higher education noted affected professional careers, leading to substantial work absenteeism. Males and younger individuals reported higher anxiety levels. A higher DLQI (>10) was reported more by men and overweight individuals.

## Figures and Tables

**Table 1:** HS patient characteristics: subject distribution according to age, sex, education level, skin phototype, hair structure, professional status, disease onset, average time to diagnosis, and most common misdiagnosis.

Variables	Patients (n = 49)	*p* Value
Age, years		
Mean ± SD	39.91 ± 13.665	0.565
Median (range)	39.24 (18.73)	
Sex		
Female, n, (%)	21 (42.85%)	0.378
Male, n, (%)	28 (57.14%)	
Education level		
Primary education, n (%)	7 (14.28%)	
Secondary education, n (%)	17 (34.69%)	
Higher education, n (%)	25 (51.02%)	
Average BMI kg/m^2^	28.44 ± 6.142	0.372
Female BMI kg/m^2^	28.12 ± 5.819	
Male BMI kg/m^2^	28.68 ± 6.142	
Waist circumference, cm	91.85 ± 16.806	0.077
Females, cm	85.90 ± 19.944	
Males, cm	96.32 ± 12.619	
Weight, kg	87.93 ± 21.025	<0.001
Females, kg	80.04 ± 24.340	
Males, kg	93.85 ± 16.497	
Overweight, n, (%)	33 (67.35%)	
Skin phototypes, n, (%)		
I	15 (30.61%)	
II	19 (38.77%)	
III	13 (26.53%)	
IV	2 (4.08%)	
Hair structure, n, (%)		
Straight	32 (65.30%)	
Wavy	13 (26.53%)	
Curly	4 (8.16%)	
Professional status, n, (%)		
Working/Student	41 (83.67%)	
Unemployed	2 (4.08%)	
Disability	5 (10.20%)	
Pensioner	1 (2.04%)	
Disease onset (years ± SD):	25.71 ± 13.743	0.425
Females	26.61 ± 13.93	0.865
Males	25.03 ± 13.743	0.243
Medical care started (years ± SD)	28.22 ± 14.000	0.391
Females	27.57 ± 14.204	0.452
Males	28.71 ± 14.003	0.841
Time to diagnosis (years ± SD)	5.2 ± 7.607	0.201
Females	6.5 ± 7.748	<0.001
Males	4.2 ± 7.607	0.540
Misdiagnosis, n, %		
Total	33 (70.2%)	
Furuncle	17 (51.51%)	
Ulcer	4 (12.12%)	
Abscess	3 (9.09%)	
Acne	2 (6.06%)	
Allergy; Complication of Diabetes; Mycosis; Lymphadenitis; Pilonidal sinus; Folliculitis; Psoriasis	1 (3.03%)	

**Table 2:** Pain caused by HS: number of painful days over the preceding 4 weeks and average VAS scores.

Variables	All Patients (n = 49)	Females (n = 21)	Males (n = 28)	*p*-Value
Painful days over preceding 4 weeks (±SD)	6.17 ± 6.98	6.61 ± 7.06	5.92 ± 6.85	0.005
Average VAS score (±SD)	5.60 ± 3.36	5.42 ± 3.34	5.74 ± 3.36	0.003
Persistent pain, n, %	15 (30.61%)	8 (38.09%)	7 (25%)	0.062
Intermittent pain, n, %	34 (69.38%)	13 (61.90%)	21 (75%)	0.003

## Data Availability

The data that support the findings of this study are available from the corresponding author upon reasonable request.
